# Supplementary material for: Incongruent Views of Functioning between Patients and Gastroenterologists: A Mixed Methods Study
Source: Healthcare (Basel). 2022 Dec 26;11(1):62. doi: 10.3390/healthcare11010062 (PMC9818837; doi:10.3390/healthcare11010062)
Supplement: Supplementary file 1 [file healthcare-11-00062-s001.zip › Supplementary Tables.zip!/Supplementary Tables.pdf]

Supplementary Table S1. Codes and Definitions for Patient Theme One: 'Illness, Emotional and Personal Problems'.

**Codes**	**Definition**
Physical-emotional problems	Fragments of narratives that illustrate that a physical illness can cause emotional problems.
Acceptance of symptoms	Fragments that refer to the acceptance of symptoms, to the incorporation of symptoms into daily life or adapting living habits due to these symptoms.
Emotional consequences	Fragments of the narratives that highlight the consequences that the digestive disorder has on the mood of the person suffering from this disorder.
Relationship-consequences	Fragments of narratives that refer to the fact that the digestive disorder affects personal relationships, as well as to the way in which the person relates to others.
Consequences-daily life	Fragments of narratives that exemplify the consequences that a digestive disorder has on daily activities (i.e. on work, leisure and travel).
Stomach upset-stressful events	Narratives that refer to a suspected link between a stressful life event and stomach discomfort.
Emotional/personal problems	Fragments of narrative from patients that refer to the existence of emotional (e.g. anxiety, depression) or personal problems (e.g. unemployment, marital problems, bereavement, medication, alcohol and drug abuse).
Experience of the disease	Fragments of narrative that indicate that each person lives with their illness or symptoms depending on how they interpret them. Additionally, this refers to whether the patient allows it to affect them or to occupy a central place in their life.

Supplementary Table S2. Codes and Definitions for Patient Theme Two: 'Disease-Healthcare System Interaction'.

**Codes**	**Definition**
Empathy towards professionals	Fragments of narratives in which patients express an understanding towards health professionals. This refers to the way in which personal problems or an excessive workload can affect the way in which doctors treat patients.
Uncertainty over waiting times	Parts of the narrative that refer to the fact that delays in medical test results or waiting periods until the medical visit causes uncertainty and concern for the patient.
Dissatisfaction with professionals	Fragments of narrative in which patients express dissatisfaction with the treatment or quality of care that they receive.
Dissatisfaction with treatment	Fragments of narratives in which patients express dissatisfaction with prescribed treatment.
Discomfort-absence of diagnosis	Fragments relating to the existence of stomach problems and discomfort without a cause after diagnostic tests have been carried out.
The need for patient strategies	Fragments in which patients express the need for tools and strategies in order to cope with the disease.
Satisfaction with professionals	Parts of the narrative that refer to the satisfaction with the treatment and quality of health care received.
Feelings of isolation/abandonment	Fragments that refer to the feeling of abandonment and lack of accompaniment by health professions and the health system in certain disease processes.
The feeling of being understood	Fragments that refer to the sensation that health professionals strive to understand the patient's discomfort and their situation.
Feeling misunderstood	Fragments that allude to the feeling that professionals do not understand the patient's situation or discomfort, they do not empathise with them or take them seriously.
Veracity of complaints	Parts that refer to the patient's feelings that professionals believe that they exaggerate about their discomfort, or do not take their discomfort seriously.

Supplementary Table S3. Codes and Definitions for Patient Theme Three: 'Health System'.

**Codes**	**Definition**
Comprehensive care	Fragments that refer to the intervention as being of an integral nature such as seeing the person as a whole, taking into account psychological aspects, and the personal life of the patient.
Lack of clarity	Parts that refer to a lack of clarity in the explanations given by professionals regarding the disease and the steps that patients have to take in order to move closer to the healing process.
Visiting time	Fragments that refer to the healthcare professional taking the time that is deemed necessary depending on the patient's needs rather than what has been stipulated
Generational change	This refers to the perception that new generations of professionals work more closely with patients, use a more suitable communicate style, and clarity in their explanations.
Communication with professionals	This outlines the need for a suitable communication style with the healthcare professional as this facilitates understanding, the diagnostic process, the search for treatment, and provides peace of mind to the patient.
Coordination between services	This refers to coordination and communication among health professionals regarding patients they have in common.
Difficulty accessing specialists	Fragments of narratives that make reference to the difficulties that patients face when requiring access to or when needing to be referred to specialists.
Not listened to enough	This refers to professionals not listening to patients and overlooking important information.
Lack of resources	The patient has given the impression that the public health system has worsened due to a lack of economic resources.
Inaccessibility of the professional	Refers to the belief that healthcare professionals feel that they are superior to patients.
Waiting lists	This refers to the time it takes to ask for a doctor's appointment until the time you have one which is considered as being too long.
Health insurance	This refers to the preference of being cared for by the Social Security system instead of using private health insurance in order to obtain the best quality of care. It also refers to the fact that when you have health insurance there are no waiting times.

Participation in studies	Parts of narratives that allude to patient involvement in research studies.
Possible negligence	Fragments of text that refer to the existence of possible medical malpractice.

Supplementary Table S4. Codes and Definitions for Physician Theme One: 'Intervention'.

**Codes**	**Definition**
Openness with the patient	Fragments of text that refer to the importance of having a clear and honest communication style when discussing the patient's disease and symptoms.
Understanding	This relates to the fragments of text that exemplify the work the professional does to understand the patient and thus help the patient better understand their illness.
Differences in explanation	Fragments of narratives that expose the need of using different strategies to explain the diagnosis for patients with functional digestive disorders due to the difficulty of understanding this disorder.
Difficulty with the diagnosis	Parts of the narrative expressing the difficulty of making a diagnosis based on the prevalence of the disease.
Difficulties with the doctor-patient relationship	Fragments that refer to the difficulty of establishing a good relationship with a patient.
Empower	Fragments of the narrative that refer to the work that healthcare professionals must do in order for the patient to take responsibility for what happens to them. Additionally, that the professional must have a passive role.
Lack of tools	Parts of the narrative referring to the lack of communication resources and tools for managing characteristics inherent to patients with functional digestive disorders.
Medical record	Fragments of the narrative that refer to the notes that professionals make in the medical records. This is in order to remind themselves of the patient's previous visits or to make it easier for their colleagues.
Importance of the diagnosis	Fragments of the narrative that considers that a good explanation, understanding and internalisation of the diagnosis by the patient is key throughout the process.
The importance of a relationship	This refers to fragments of text outlining the importance of having a good relationship between the doctor and

	the patient. This needs to be based on trust, which is especially important in the intervention process for patients with functional digestive disorders.
Comprehensive intervention	Reflects the need and importance of treatment that integrates physical, psychological and personal aspects.
The need for more time with patients with FGIDs	Fragments of narratives in which professionals express that more time is needed when working with patients with functional digestive disorders as it is important to give them a good explanation of their pathology and create a good doctor-patient bond.
Reassurance	The fragments of text that refer to the part of the intervention that involves reassuring patients by explaining to them that they do not suffer from a life-threatening illness.

Supplementary Table S5. Codes and Definitions for Physician Theme Two: 'Patients'.

Codes	Definition
Adherence to treatment	Fragments of narrative which outline the importance for patients to follow guidelines and treatment prescribed by the doctors. This also refers to the difficulties doctors face in ensuring that patients keep to it.
Differentiating characteristics	These fragments exemplify the characteristics of digestive disorders, as well as patients who have digestive disorders. This leads to differences to other types of disorders when communicating and carrying out interventions with this subgroup of patients.
Disease awareness	Fragments of narrative highlighting the need to raise awareness of the presence of the disorder and the importance of following a treatment.
Mistrust	Parts of the narrative in which the mistrust that the patients have is felt by the doctors. This mistrust is either towards the doctors themselves, or towards the diagnosis or medication which is being given to them.
Gender difference	Fragments of text that refers to the differences observed by professionals between men and women in terms of adherence to treatment.
Brain-gut axis	Fragments of narratives that refer to the implications of the intervention on patients with functional digestive disorders and the fact that two different systems intervene.
Importance of psychological aspects	Parts of the narrative that refer to the influence of emotional discomfort (i.e. stress, anxiety, depression, personal situations) on digestive problems.
Importance of sociodemographic characteristics	Fragments of the narrative that refer to the importance of sociodemographic characteristics and the patient's history in explaining the disease, treatment, and to be able to intervene appropriately.
Non-acceptance of diagnosis	Fragments of the narrative exposing the difficulty with which patients find themselves accepting the FGID diagnosis due to its characteristics and the chronicity of it.

Personality pattern	This refers to fragments of text that outline the existence of a specific personality pattern and a way of relating to the environment for patients with FGIDs.

Supplementary Table S6. Codes and Definitions for Physician Theme Three: 'Health system'.

Codes	Definition
Lack of mental health referral resources	Fragments of text that outline the difficulties that physicians face when referring patients with mental health problems to mental health services.
GP	Fragments of narrative reflecting the importance of the GP's support, as they know the patient and family best.
Biomedical models-chronic disease	Fragments of narratives exposing the need of changing the paradigm from a biomedical model to a biopsychosocial model as it is more suited to the needs of patients with chronic diseases.
The need for a psychologist	Fragments of narratives exposing the need for a primary care psychologist due to the prevalence of emotional problems that have been observed in patients.
Overload-consequences	Fragments of text that refer to the consequences of having an excess of patients and the effect that this has on the quality of care.
